# Efficacy of Willow Herb (*Epilobium angustifolium* L. and *E. parviflorum* Schreb.) Crude and Purified Extracts and Oenothein B Against Prostatic Pathogens

**DOI:** 10.3390/antibiotics14020117

**Published:** 2025-01-23

**Authors:** Alexia Barbarossa, Antonio Rosato, Alessia Carocci, Sabrina Arpini, Stefania Bosisio, Luca Pagni, Diletta Piatti, Eleonora Spinozzi, Simone Angeloni, Gianni Sagratini, Gokhan Zengin, Marco Cespi, Filippo Maggi, Giovanni Caprioli

**Affiliations:** 1 Department of Pharmacy-Pharmaceutical Sciences, University of Bari “Aldo Moro”, Via E. Orabona, 4, 70125 Bari, Italy; alexia.barbarossa@uniba.it (A.B.); antonio.rosato@uniba.it (A.R.); alessia.carocci@uniba.it (A.C.); 2 Indena S.p.A., Via Don Minzoni 6, 20090 Settala, Italy; sabrina.arpini@indena.com (S.A.); stefania.bosisio@indena.com (S.B.); luca.pagni@indena.com (L.P.); 3 Chemistry Interdisciplinary Project (ChIP) Research Center, School of Pharmacy, University of Camerino, Via Madonna delle carceri, 62032 Camerino, Italy; diletta.piatti@unicam.it (D.P.); eleonora.spinozzi@unicam.it (E.S.); simone.angeloni@unicam.it (S.A.); gianni.sagratini@unicam.it (G.S.);; 4 Department of Biology, Science Faculty, Selcuk University, Konya 42130, Turkey; gokhanzengin@selcuk.edu.tr

**Keywords:** *Epilobium angustifolium*, *Epilobium parviflorum*, prostatic pathogens, HPLC-MS/MS, oenothein B, gram-positive

## Abstract

**Background/Objectives**: Plants species of the *Epilobium* genus are traditionally used to treat prostatitis and other urinary tract disorders and are particularly rich in ellagitannins and flavonol 3-*O*-glycosides. The aim of this work was to evaluate the inhibitory activity of different extracts from *E. angustifolium* L. and *E. parviflorum* Schreb. and their major bioactive compound, oenothein B, against a panel of Gram-positive (*Enterococcus faecalis* ATCC 29212, *Enterococcus faecalis* BS, *Staphylococcus aureus* ATCC 25923, *Staphylococcus aureus* ATCC 29213, and *Staphylococcus aureus* ATCC 43300) and Gram-negative (*Escherichia coli* ATCC 25922, *Escherichia coli* ATCC 35218, *Escherichia coli* BS, *Klebsiella pneumoniae* ATCC 13883, *Klebsiella pneumoniae* ATCC 70063, *Klebsiella pneumoniae* BS, *Proteus mirabilis* BS, and *Pseudomonas aeruginosa* ATCC 27853) bacteria responsible for prostatitis. **Methods**: Aqueous and ethanolic raw extracts were prepared, and the latter were further purified using the resin Amberlite^TM^ XAD7HP. Then, an HPLC-MS/MS method was developed for the quantification of the marker bioactives and their levels were correlated with the antimicrobial activity. **Results**: Purified extracts were richer in polyphenols (330.80 and 367.66 mg/g of dry extract for *E. angustifolium* and *E. parvifolium*, respectively) than the raw extracts. Oenothein B was the predominant compound in all the extracts (119.98 to 327.57 mg/g of dry extract). The minimum inhibitory concentrations (MICs) in the range of µg/mL indicated significant antibacterial activity, which was higher for the purified extracts and oenothein B (MIC values from 4 to 16 and 8 to 1024 µg/mL on Gram-positive and Gram-negative strains, respectively). **Conclusions**: These results outline the outstanding potential of *E. angustifolium* and *E. parviflorum* extracts and oenothein B as therapeutic alternatives or complementary agents to conventional antibiotic treatments of prostatitis and other urinary tract infections.

## 1. Introduction

The genus *Epilobium*, belonging to the Onagraceae family, encompasses 200 species, mostly erect perennial herbs, distributed around the world, with twenty-six species occurring in Europe [1]. One of the most important traditional uses of these plants, mainly in Europe and North America, consists in the treatment of skin and mucosa infections and disorders of the genito-urinary tract such as prostatitis, with the latter being characterized by inflammatory process and/or benign prostatic hyperplasia (BPH) [2,3]. The marker compounds of the *Epilobium* genus responsible for the therapeutic properties (e.g., anti-inflammatory and antioxidant) are phenolics, mostly ellagitannins (oenothein B), and flavonol 3-*O*-glycosides (kaempferol, quercetin, and myricetin derivatives) [2,4,5,6].

BPH is a common human disease affecting men over 50 years [7], and its timely treatment is of pivotal importance to avoid recrudescence of the pathology leading to tumor formation [8]. Therapeutic preparations used in the treatment of prostatic disorders based on *Epilobium* species are mostly characterized by *E. angustifolium* L. and *E. parviflorum* Schreb. extracts [1,9]. Their use in such preparations is supported by studies that have investigated the safety profile and toxicity of these extracts, highlighting their low toxicity and safety at moderate doses [10,11]. *E. angustifolium*, also known as fireweed, is a perennial herb native to Northern and Eastern Europe, where it has been traditionally used as a wound-healing and anti-infective agent and for the treatment of prostatic disorders [12]. In 2019, the European Medicines Agency (EMA) claimed the traditional use of preparations made up of aerial parts as a reliever for urinary tract disorders with a particular efficacy towards BPH [13]. *E. parviflorum*, also known as small-flower willowherb, is distributed in Europe, Western Asia, North Africa, and North America. In traditional Chinese Medicine, *E. parviflorum* has been used for the treatment of inflammations and menstrual disorders [14]. In Europe, preparations made up of its aerial parts, which are approved by the European Medicinal Agency (EMA), are used for the treatment of prostatic and urinary tract disorders [11].

The efficacy of *E. angustifolium* and *E. parviflorum* extracts on prostatic ailments relies on modulation of androgens, inhibition of PSA (prostatic specific antigen) and metalloproteinases, and antiproliferative and pro-apoptotic activities [1].

Acute bacterial prostatitis is caused by opportunistic bacteria colonizing the urinary tract following an infection or obstruction such as *Escherichia coli* and others [15]. Acute bacterial prostatitis is an acute infection of the prostate gland that causes pelvic pain and urinary tract symptoms, such as dysuria, urinary frequency, and urinary retention, and may lead to systemic symptoms, such as fevers, chills, nausea, emesis, and malaise. While the exact prevalence is unclear, it is estimated that acute bacterial prostatitis accounts for about 10% of all prostatitis cases [16]. The infection typically arises from bacteria that ascend through the urethra, the backward flow of contaminated urine into the prostatic ducts, direct bacterial introduction during procedures like transrectal biopsy or urethral instrumentation, or through hematogenous spread [17]. Most cases (65–80%) are caused by *E. coli*, while other pathogens such as *Pseudomonas aeruginosa*, *Proteus mirabilis*, *Klebsiella* spp., and *Enterobacter* spp. account for the rest [18,19]. In individuals with compromised immunity, additional pathogens, including fungi like *Cryptococcus* spp. or *Histoplasma* spp., may also play a role in the infection [20,21]. The cure for bacterial prostatitis is hindered by the lack of a transport mechanism of antibiotics as well as by their scarce penetration into the prostatic tissue [22]. Only selected antibiotics are effective for treating bacterial infections of the prostate. While most antibiotics can penetrate the acutely inflamed prostate, this is not the case for a chronically inflamed gland. The capillaries in the prostate are nonporous and lack a mechanism for antibiotic transport. Fluoroquinolones possess the best pharmacological properties for treating bacterial prostatitis, achieving concentrations in the prostate that are 10 to 50% of those found in the serum [23]. Clindamycin, azithromycin, trimethoprim-sulfamethoxazole, and doxycycline are antibiotics encompassed with good penetration capacity into the tissues, as well as carbapenems, piperacillin, cephalosporins, and some of the aminoglycosides [24]. However, one of the major threats in managing bacterial prostatitis is the growing resistance of microorganisms, especially to fluoroquinolones. Indeed, since fluoroquinolones have a broad spectrum of activity, their use has increased, leading to resistance [25]. Hence, given the increasing prevalence of drug-resistant strains to conventional therapies, the study of new, effective antibacterial agents to combat bacterial prostatitis is an urgent priority. In this context, natural extracts, with their vast array of phytochemicals, offer a valuable resource for discovering novel compounds that could overcome these therapeutic challenges and provide sustainable solutions to this concern.

To the best of our knowledge, the activity of *E. angustifolium* and *E. parviflorum* extracts against opportunistic bacteria occurring during prostatic disorders has not been fully explored. So far, the inhibitory properties of *E. angustifolium* extracts against reference strains from collection associated with skin infections such as *Streptococcus pneumoniae*, *E. coli*, *Enterococcus faecalis*, *E. faecium*, *Sarcina lutea*, and *Bacillus pseudomycoides* have been evaluated [26].

Therefore, in an attempt to support the traditional uses of these species as anti-infective agents useful in treating urinary tract-related disorders, and to deepen knowledge of their antibacterial spectrum, in this work, we evaluated the inhibitory properties of *E. angustifolium* and *E. parviflorum* polar extracts against a panel of bacterial strains both derived from clinical isolation and ATCC collection, which are correlated with opportunistic infections of the urinary tract. To enhance the antibacterial potency, plant extracts were subjected to a purification procedure. An HPLC-MS/MS method was developed and used for the quantitative determination of fifteen bioactive compounds in *Epilobium* extracts, among which oenothein B was used as a quality marker and to correlate their levels with the antibacterial activity observed.

## 2. Results

### 2.1. HPLC-MS/MS Analysis

#### 2.1.1. Optimization of the HPLC-MS/MS Analytical Conditions

An HPLC-MS/MS analysis was performed in dynamic-MRM mode, permitting us to simultaneously monitor the transitions of all the analytes according to their retention times. The MS/MS transitions of each analyte were optimized in flow injection analysis (FIA) using MassHunter Optimizer Triple Quad ver. B.04.01 software (Agilent, Santa Clara, CA, USA) through the injection of an individual standard solution (10 μg/mL) for each analyte. The precursor ions of all analytes except for oenothein B were the deprotonated molecules [M − H]^−^ in negative polarity. Instead, the precursor ion of oenothein B was the molecule minus two protons, [M − 2H]^2−^, as reported in Table 1 and in Figures S1–S4 of the Supplementary Materials.

**Figure 1:**
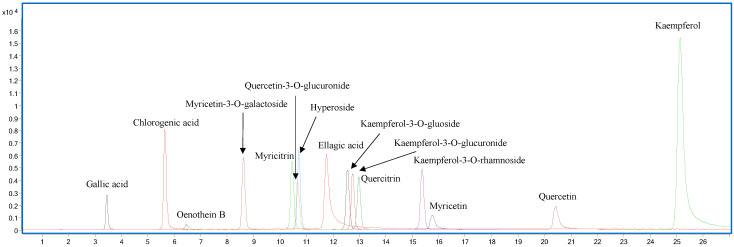
Overlay of all the chromatograms obtained using the extraction of the quantitative transition (dynamic-MRM) of each analyte of the standard mixture.

Figure 1 reports the overlay of all chromatograms of the standard mixture obtained by the extraction of the quantitative transition of each analyte.

Several attempts have been carried out to optimize the analyte separation. In detail, two analytical columns, i.e., Kinetex PFP (100 × 2.1 mm, particle size 2.6 μm) and Sinergy Polar-RP 80A (150 mm × 4.6 mm, 4 μm), both from Phenomenex (Castel Maggiore, Bologna, Italy), were evaluated. With the Sinergy Polar column, a better resolution and analyte separation were achieved. Moreover, the shape and the width of the oenothein B peak were preferable to that resulting from the Kinetex PFP column. In addition, different mobile phases (water/methanol, water/acetonitrile both with/without formic acid) and several gradients were evaluated to optimize the separation of the analytes. Table S1 (Supplementary Materials) summarises the best gradients obtained, all with water/acetonitrile and formic acid. The best-performing one in terms of resolution and analyte separation was Gradient 4. This one was selected for the present study.

#### 2.1.2. Method Validation

The analytical method validation was assessed in terms of linearity, limit of detection (LOD), limit of quantification (LOQ), and repeatability. Linearity was evaluated by constructing calibration curves with the respective determination coefficients (R^2^) after the injection of different concentrations of the analytes. All the R^2^ ranged from 0.998 to 1.000, displaying good linearity. LOD was calculated as the lowest standard concentration with a signal-to-noise ratio (SNR) = 3, whereas LOQ with SNR = 10 (Table 2).

RSD% was employed to express the intraday repeatability or run-to-run precision and inter-day repeatability or day-to-day precision. Run-to-run precision ranged from 0.02 to 8.26%, whereas day-to-day precision was from 0.81 to 12.73%. High specificity was achieved using HPLC-MS/MS in dynamic-MRM mode. Method specificity was assessed by measuring retention time stability and setting up multiple precursor/product pairs.

#### 2.1.3. Quantification of Phenolic Compounds

The validated HPLC-MS/MS method was applied for the quantification of fifteen phenolic compounds in the extracts of *E. angustifolium* and *E. parviflorum*. In detail, three diverse extracts were analyzed for each *Epilobium* species, namely, RE1_ang_/RE1_parv_, RE2_ang_/RE2_parv_, and PE_ang_/PE_parv_. The results obtained for the quantification of the marker polyphenols are reported in Table 3.

Generally, all REs displayed a comparable polyphenol content, while the PEs resulted in particularly enriched targeted compounds. Indeed, the total amount of polyphenols was 154.74 and 192.50 mg/g of dry extract for RE2_ang_ and RE2_parv_, respectively. Conversely, the PEs were enriched in polyphenols, with a content of 330.80 and 367.66 mg/g of dry extract for *E. angustifolium* and *E. parviflorum*, respectively. This result demonstrates the crucial importance of the purification process for the removal of undesired macromolecules and sugars and to produce enriched bioactive compounds. Both *E. angustifolium* and *E. parviflorum* PEs were dominated by high levels of oenothein B accounting for 255.86 and 327.57 mg/g of dry extract, respectively.

Regarding the other polyphenols, some differences between the two species were detected. In detail, *E. angustifolium* PE was characterized by higher contents of quercetin-3-*O*-glucuronide (42.08 mg/g of dry extract), chlorogenic acid (6.34 mg/g of dry extract), hyperoside (6.09 34 mg/g of dry extract), and kaempferol-3-*O*-glucuronide (5.42 mg/g of dry extract). On the other hand, *E. parviflorum* PE displayed a higher content of ellagic acid (11.54 mg/g of dry extract), myricetin-3-*O*-galactoside (10.16 mg/g of dry extract), myricitrin (9.56 mg/g of dry extract), and chlorogenic acid (2.57 mg/g of dry extract).

### 2.2. Antibacterial Activity

The presented study focused on the evaluation of the antibacterial activity of the different extracts against the strains that are the main culprits of bacterial prostatitis with the aim of finding safe therapeutic alternatives to overcome the problem of antibiotic resistance. The antibacterial activity of *E. parviflorum* and *E. angustifolium* extracts, along with their predominant compound oenothein B, was investigated following the CLSI guidelines to determine the Minimum Inhibitory Concentration (MIC) and Minimum Bactericidal Concentration (MBC) against various Gram-positive and Gram-negative bacterial strains (M07-A9, 2012). These strains comprised both those from the American Type Culture Collection (ATCC) and clinical isolates. The results demonstrated remarkable antibacterial activity of both botanical species against the tested strains (Table 4). Notably, MIC values were observed in the range of µg/mL, indicating significant potency. In some instances, the MIC values were comparable to those of the reference antibiotic used in the assay, highlighting the potential efficacy of these extracts. Moreover, no remarkable differences were observed between extracts from the two species. However, PEs of both species exhibited the highest antibacterial activity, with MICs of 4 µg/mL against *Staphylococcus aureus* ATCC 25923 and *S. aureus* ATCC 29213, and 8 µg/mL against the MRSA strain *S. aureus* ATCC 43300. Additionally, *E. parviflorum* PE showed a particularly interesting effect against *E. faecalis* ATCC 29212 and clinical isolate *E. faecalis* BS, with MICs that were sometimes comparable to those of levofloxacin. The MIC values for all extracts against Gram-negative bacteria ranged from 8 to 1024 µg/mL, thus indicating a wide variability depending on the strain considered. Remarkably, PEs of both species displayed a noteworthy activity against the pathogen *P. aeruginosa*, which was identified as the most sensitive Gram-negative strain, with a determined MIC of 8 µg/mL (Table 4). This result is extremely interesting as *P. aeruginosa* is a complex nosocomial pathogen, highly resistant to conventional therapies, and difficult to eradicate. It is worthy of note that the MBC values differed by only one dilution from the MICs, demonstrating the bactericidal action of these natural extracts. Overall, it is possible to observe that there was variability in activity depending on the extraction method and bacterial strain. In fact, the PEs resulted in much more active than REs towards the strains under study, highlighting the importance of purification methods in enhancing the content of bioactive compounds, especially oenothein B, and thus increasing the antibacterial properties of the plants. To support this evidence, the antibacterial effects of oenothein B alone have been investigated. Oenothein B demonstrated potent activity against Gram-positive strains, with MIC values consistently lower than or comparable to those of the extracts. For example, against *E. faecalis* ATCC 29212, oenothein B exhibited a MIC of 8 µg/mL, while the MIC values for RE1*_ang_* and RE2*_ang_* were 16 µg/mL. Similarly, for *S. aureus* ATCC 29213, the MIC of oenothein B (4 µg/mL) was lower than the MIC values of both raw and purified extracts (8–16 µg/mL); thus, the high content of oenothein B in the purified extracts appears to be fundamental to their antimicrobial activity, particularly against Gram-positive bacteria. In contrast, as pointed out for the extracts, Gram-negative strains exhibited lower susceptibility to oenothein B, with MIC values ranging from 64 µg/mL (*K. pneumoniae* BS) to 1024 µg/mL (*E. coli* BS). These values were like or slightly lower than those observed for the crude extracts. For instance, against *E. coli* ATCC 25922, the MIC of oenothein B (256 µg/mL) was identical to that of RE1*_ang_* and RE2*_ang_*. Indeed, it has been found a negative very strong (r > 0.9) or strong Pearson correlation (0.7 < r < 0.89) between oenothein B concentration and the MIC values for some specific strains such as *S. aureus* ATCC 25923, *S. aureus* ATCC 29213, *E. coli* BS, and *K. pneumoniae* BS (Table 5). While the comparable MIC values of oenothein B and the crude extracts against Gram-negative strains suggest a contribution from other phytochemical components or synergistic effects, the results underline the pivotal role of oenothein B as the primary active compound. However, the results achieved for Gram-negative strains suggest a potential synergistic effect of oenothein B with other minor components present in the extracts.

## 3. Discussion

The high levels of oenothein B detected for the two species, corresponding to 25 and 32% of dry extracts, respectively, were significantly higher than those reported in the literature for *Epilobium* extracts, which vary from 2 to 14% [2]. Thus, the purification procedure applied resulted in an added value for the enhancement of the biological activity of these extracts. The data obtained in this work are in accordance with those previously published. Indeed, oenothein B has been reported as the main polyphenol found in *E. angustifolium* and *E. parviflorum*, even if its levels vary according to different factors, such as flowering stage or geographic origin [5,27,28,29,30]. For instance, Kiss et al. [5] reported content of the compound of 225.8 and 326.7 mg/g for *E. angustifolium* and *E. parviflorum* dry aqueous extracts, respectively. These concentrations are slightly higher than those reported in the aqueous extracts analyzed in this work. Conversely, in the work of Baert et al. [29], the level of oenothein B in *E. angustifolium* methanolic extract ranged from 24.90 to 50.26 mg/g of dry extract, quite lower than that reported in this work. Concerning the other polyphenols, for *E. angustifolium*, the main polyphenols reported were also quercetin-3-*O*-glucuronide and gallic acid together with kaempferol-3-*O*-glucuronide [27]. Indeed, quercetin-3-*O*-glucuronide and kaempferol-3-*O*-glucuronide have been identified as chemotaxonomic markers of *E. angustifolium* [6,11]. On the contrary, besides oenothein B, ellagic acid has been reported as one of the main polyphenols of *E. parviflorum* [31], as myricetin-3-*O*-galactoside [30].

Bacterial prostatitis is an inflammatory condition of the prostate gland primarily caused by bacterial infections. The main strains responsible for this condition include *E. coli*, which is the most common pathogen associated with acute bacterial prostatitis, as well as other Gram-negative bacteria such as *K. pneumoniae* and *P. mirabilis* [32]. Additionally, Gram-positive bacteria, including *E. faecalis* and *S. aureus*, may also contribute to chronic forms of the diseases [33]. The antibacterial properties of various natural products used alone or in synergy with conventional antibiotic or non-antibiotic drugs [34] have garnered significant attention in recent years, particularly considering the increasing prevalence of antibiotic-resistant pathogens. Indeed, the understanding of the antibacterial properties of various natural extracts could provide alternative therapeutic options for managing bacterial infections, among which is prostatitis. Among the plant extracts, the genus *Epilobium* has emerged as a promising source of antimicrobial compounds [35]. Specifically, *E. angustifolium* and *E. parviflorum* have been the subjects of numerous studies, revealing their potent antibacterial activities against several strains of clinical interest [36,37]. This paper explored the antibacterial activities of crude and purified extracts from both *E. angustifolium* and *E. parviflorum*, contributing to the growing body of evidence supporting their medicinal potential. However, several studies have previously explored the antibacterial effects of *Epilobium* extracts, considering different Gram-positive and Gram-negative strains. Studies on *E. parviflorum* demonstrated its effectiveness against *P. mirabilis* (ATCC 21721), with methanolic and ethyl acetate extracts exhibiting MICs of 484 and 623 µg/mL, respectively, and the aqueous extract displaying moderate activity with an MIC of 1834 µg/mL [38]. These findings are consistent with our observations on the clinical isolate *P. mirabilis* BS, which exhibited sensitivity to both extracts. However, extracts obtained using ethanol as a solvent demonstrated superior activity against this strain (MIC values amounting to 32 µg/mL). Furthermore, *E. angustifolium*, on the other hand, has demonstrated a broader antibacterial activity. Indeed, significant differences in the MICs between strains were detected for *E. coli* (from 0.625 µg/mL to 16.2 mg/mL), *S. aureus* (from 0.625 µg/mL to 7.6 mg/mL), and *P. aeruginosa* (from 1.25 µg/mL to 9.1 mg/mL) [39,40,41,42]. These differences are mainly due to the method of extraction, the part of the plant used, and, consequently, the variability of the components contained in the extracts. Ellagitannins are compounds with documented antimicrobial properties [42,43,44,45]. Some of these compounds, including oenothein B, also displayed a synergistic effect with currently used antibiotics against drug-resistant bacteria, such as *S. aureus*. For instance, the combination of oenothein B and tellimagrandin I with oxacillin resulted in a significant lowering of the MICs of the 1/250 or 1/500 antibiotics [46]. Moreover, concerning the mechanism of action, these compounds, including oenothein B, are thought to disrupt bacterial membranes, leading to increased permeability and leakage of cellular contents [47,48]. In addition, several studies corroborated their ability to bind to proteins through non-covalent bonds, which may neutralize bacteria by interfering with their membrane proteins [49]. The other mechanisms proposed for antibacterial activity include the inhibition of extracellular microbial enzymes, oxidative phosphorylation, and disruption of cellular membrane permeability [50,51]. Moreover, some of the compounds present in the extracts may act as efflux pump inhibitors, enhancing the efficacy of antibiotics by preventing bacterial cells from expelling these drugs [38]. Thus, the high content of oenothein B in all tested extracts, particularly in PEs, could justify the promising antibacterial activity detected in this study.

The results presented with this study underscore the importance of exploring natural products for their therapeutic potential. Indeed, based on our findings, it is uncommon to encounter natural substances with a high level of effectiveness against resistant pathogens, especially those demonstrating an activity comparable to that of widely used antibiotics. This aspect outlines the potential value of *E. angustifolium* and *E. parviflorum* as therapeutic alternatives or complementary agents to conventional antibiotic treatments. Therefore, future studies could be directed at investigating a possible synergistic effect of these extracts with classical antibiotics to enhance their effect, decrease toxicity, and overcome antibiotic resistance.

## 4. Materials and Methods

### 4.1. Chemicals and Reagents

Gallic acid (CAS Number 149-91-7), chlorogenic acid (CAS Number 327-97-9), oenothein B (CAS Number 104987-36-2), myricitrin (CAS Number 17912-87-7), quercetin-3-*O*-glucuronide (CAS Number 22688-79-5), hyperoside (CAS Number 482-36-0), ellagic acid (CAS Number 476-66-4), kaempferol-3-*O*-glucoside (CAS Number 480-10-4), quercitrin (CAS Number 522-12-3), myricetin (CAS Number 529-44-2), kaempferol-3-*O*-rhamnoside (CAS Number 482-39-3), quercetin (CAS Number 117-39-5), kaempferol (CAS Number 520-18-3), and oenothein B were purchased from Merck (Milan, Italy). Myricetin-3-*O*-galactoside (CAS Number 15648-86-9) and kaempferol-3-*O*-glucuronide (CAS Number 22688-78-4) were purchased from Extrasynthese (Genay, France). Individual stock solutions of each analyte, at a concentration of 1000 µg/mL, were prepared by dissolving pure standard compounds in HPLC-grade methanol:water (80:20) and storing them in glass-stoppered bottles at 4 °C. Regarding ellagic acid, the stock solution was prepared by dissolving 5 mg in 25 mL of an HPLC-grade acetone:acetonitrile:ethanol mixture (1:1:1). HPLC-grade methanol was purchased by Carlo Erba (Milan, Italy), while HPLC-grade formic acid (99%) was acquired from Merck (Darmstadt, Germany), as well as the resin Amberlite^TM^ XAD7HP. A Milli-Q SP Reagent Water System (Millipore, Bedford, MA, USA) was employed to produce ultrapure water. All the solutions were filtered with Phenex™ RC 4 mm 0.2 µM syringeless filters (Phenomenex, Castel Maggiore, Italy) prior to the analysis.

### 4.2. Plant Collection and Extract Preparation

*E. angustifolium* and *E. parviflorum* aerial parts were harvested from cultivated plants near Piacenza (Italy), the former and from wild growing plants in northeastern Hungary the latter, during the pre-flowering and flowering phase, respectively (May–July 2022) Then, the plant material, deprived of the damaged and foreign parts, was air dried and subsequently milled and poured through an electric grid to obtain a homogeneous material. The botanical identification of the two species was carried out at Indena’s Botanical Laboratory, according to an internal ID method. Herbarium specimens are kept by the same lab, with protocol n. 5780 (*E. angustifolium*) and n. 5781 (*E. parviflorum*).

For the study, different extracts for each species of *Epilobium* were prepared, namely, two raw extracts (RE1*_ang_*/RE1*_parv_* and RE2*_ang_*/RE2*_parv_* for *E. angustifolium* and *E. parviflorum*, respectively) and one purified extract (PE*_ang_*/PE*_parv_* for *E. angustifolium* and *E. parviflorum*, respectively). In detail, the REs were prepared from the aerial parts (500 g) of both *Epilobium* species performing an extraction with a percolator with water at 70 °C (RE1) or ethanol 60% *v*/*v* at 25 °C (RE2) [52] covering with 3 L of solvent keeping in static conditions for at least 3 h. After discharging, additional extractions of about 1.5 L of solvent were repeated in the same conditions until complete exhaustion of the biomass (10 L of solvent employed in both cases). The resulting solutions were filtered and concentrated for dryness under vacuum using a rotary evaporator (to obtain 132 g of RE1*ang*, 134.5 g of RE1*par* and RE2*ang*, and, finally, 112 g of RE2*par*). The extraction yield (% *w*/*w*) was calculated on a dry weight basis. The extraction yields are reported in Table 6.

The purification was conducted only on ethanolic extracts since losses in oenothein B content have been observed for aqueous extracts after the clarification process. Moreover, the purification process of the aqueous extracts also led to precipitation issues in the purification column. Regarding PE*_ang_* and PE*_par_*, they were obtained via the purification of the RE2*_ang_* and RE2*_parv_* through an adsorption resin, namely, Amberlite™ XAD7-HP loading the extract in an aqueous solution to adsorb the nonpolar molecules and eliminating the more polar fraction of the total extract. The resin was selected after a screening of ten adsorbent resins (aliphatic and aromatic) with different characteristics (porosity and surfaces) due to its good capacity and extract profile. In detail, the aerial parts of the two *Epilobium* species (700 g) were extracted with ethanol 60% at 25 °C, obtaining the hydroalcoholic solutions containing the REs (about 190 g), which were concentrated until aqueous solution (about 1200–1300 g), clarified using centrifugation to separate a precipitate (with a dry residue of about 20 g containing only small amount of oenothein B) to be then loaded at a flow rate of 1.5 BV/h into a column filled with 1500 mL of Amberlite™ XAD7-HP sorbent resin previously conditioned in water. The initial aqueous eluate was discarded, and then the column was washed with 3000 mL of water. The aqueous eluates (total dry residue of about 100 g) were discarded. Then, the column was eluted with 4500 mL of 70% ethanol to desorb the PEs. The hydroalcoholic eluate was collected, concentrated to dryness under vacuum using a rotary evaporator, and dried at 50 °C under vacuum to yield (% *w*/*w*) the Pes (70 g PE*_ang_* and 63 g PE*_par_*). Table 6 reports the yield obtained for the preparation of the extracts.

### 4.3. HPLC-MS/MS Analysis

#### 4.3.1. Sample Preparation

The dry extracts RE2*_ang_*/RE2*_parv_* and PE*_ang_*/PE*_parv_* were dissolved in methanol:water (80:20) (1000 μg/mL) and sonicated for 10 min at room temperature. Conversely, the dry extracts RE1*_ang_*/RE1*_parv_* were dissolved in methanol:water (50:50) (1000 μg/mL). Then, the solutions were filtered through a 0.2 µm filter before HPLC-MS/MS analysis. All samples were stored at −15 °C prior to the analysis. Each sample was analyzed in duplicate.

#### 4.3.2. Analytical Conditions

The quantification of bioactive compounds was carried out using a 1290 Infinity series liquid chromatograph (Agilent, Santa Clara, CA, USA) coupled with a 6420 Triple Quadrupole (Agilent). The mass spectrometer was furnished with an electrospray ionization (ESI) source operating in negative ionization mode. The analytes were separated using a Synergi Polar-RP 80 Å (150 mm × 4.6 mm i.d., 4 µm) column (Phenomenex, Torrance, CA, USA). The separation was performed in gradient mode using water (A) and acetonitrile (B), both with formic acid 0.1%, as mobile phase. The flow was 0.8 mL min^−1^ and the injection volume 2 µL. The column was maintained at 25 °C, while the drying gas temperature in the ionization source was 350 °C. The gas flow was 12 L/min, the nebulizer pressure was 45 psi, and the capillary voltage was set at 4000 V. Detection was achieved in dynamic-MRM mode. The quantification was performed through the integration of the dynamic-MRM peak areas, employing the most abundant transitions of the analytes. The mass spectrometer parameters, including Rt and ΔRt, are reported in Table 1.

#### 4.3.3. Method Validation

The analyses of the solutions were performed in duplicate (n = 2). The relative standard deviation percentage (RSD %) was calculated to assess data precision. The HPLC-MS/MS method was validated by the linearity, limit of detection (LODs), limit of quantification (LOQs), and precision. The linearity was assessed by injecting standard solutions at various concentrations of the compounds mentioned in Section 2.1. The calibration curves were obtained by plotting the analyte peak areas on the analyte concentrations, and they were employed for the quantification of the marker polyphenols. The method was also assessed in terms of repeatability injecting each standard solution 3 times in the same day (intraday), as well as 3 times in 3 consecutive days (inter-day). The repeatability was evaluated in terms of relative standard deviation (RSD %). In addition, LOD was evaluated, accepting a signal-to-noise ratio (S/N) of 3:1, while the LOQ was estimated, accepting a signal-to-noise ratio of 10:1.

### 4.4. Antimicrobial Assays

The in vitro minimum inhibitory concentrations (MICs, µg/mL) and the minimum bactericidal concentration (MBCs, µg/mL) were determined by using the broth microdilution method according to the Clinical and Laboratory Standards Institute (CLSI) guidelines [53]. The concentration of the six extracts and oenothein B under investigation was initially prepared at the highest value to formulate stock solutions. These stock solutions were subsequently diluted at a ratio of 1:10 using Cation-Adjusted Mueller Hinton Broth (Oxoid, Milan, Italy). This procedure resulted in a gradient of concentrations ranging from 2048 to 2 µg/mL within the wells, achieved by performing two-fold serial dilutions in the designated testing medium. The stock solutions were prepared using pure DMSO as the diluent. We used the following bacterial strains from the American Type Culture Collection (ATCC, Rockville, MD, USA) that were available as freeze-dried discs: *E. faecalis* (ATCC 29212), *S. aureus* (ATCC 25923, ATCC 29213, ATCC 43300), *E. coli* (ATCC 25922), *K. pneumoniae* (ATCC 13883. ATCC 70063), *P. aeruginosa* (ATCC 27853), and some strains derived from clinical isolation: *E. faecalis* BS, *S. aureus* BS, *E. coli* BS (ESBL), *K. pneumoniae* BS, and *P. mirabilis* BS.

The clinical isolates were obtained from patients of the Department of Biomedical Science and Human Oncology intensive care unit, University of Bari, Italy. Established physiological and morphological methods (API systems: API 20S, API RapidStaph, API Rapid 20E) were employed for isolation and identification procedures, which were performed in the Hygiene Section of the Department. To maintain the integrity of microbial cultures and ensure their reproducibility, cryovials containing all bacterial strains were established in a suitable culture medium and subsequently stored at −80 °C. Pre-cultures for each bacterial strain were prepared using Mueller Hinton Broth (MHB) and incubated from 3 to 5 h at 37 °C. Following the CLSI M7-A9 guidelines, the turbidity of the bacterial cell suspension was adjusted to match the 0.5 McFarland Standard using a spectrophotometer (OD_625_ nm readings between 0.08 and 0.10). This standardized suspension was then diluted further (1:100) using MHB to achieve a concentration of 1–2 × 10^6^ (colony-forming units) CFU/mL. Aliquots of 200 µL from the final inoculum were introduced into each well. Control wells containing only the inoculated broth were also prepared to monitor bacterial growth. The plates were incubated at 37 °C for 24 h, with the minimum inhibitory concentration (MIC) values determined as the lowest concentration of the compounds at which no visible bacterial growth was observed [53]. Each experiment was performed three times in duplicate. Levofloxacin (Sigma Aldrich, Milan, Italy) was used as the standard reference antibiotic for this study. Moreover, the antibacterial efficacy of DMSO alone (which served as a solvent for the studied extracts) was assessed; hence, solvent volumes below 5% concentration were utilized throughout the experiments and did not affect microbial growth [54]. To determine the MBC, bacterial cultures treated with the tested compounds at varying concentrations were incubated as described in the MIC assay. Following incubation, 10 µL of each well that showed no visible bacterial growth were plated onto fresh Mueller–Hinton agar plates. The plates were then incubated at 37 °C for 24 h. The MBC was defined as the lowest concentration of the compound at which no visible bacterial growth was observed on the agar plates, indicating that ≥99.9% of the initial bacterial population had been killed. All experiments were performed in triplicate to ensure reproducibility.

### 4.5. Statistical Analysis

A Pearson correlation analysis has been carried out to highlight possible relationships between compound concentrations and MIC values for the different bacterial strains. When correlation coefficients were found to be statistically significant, the interpretation of their absolute magnitude was reported according to the following cut-off (absolute value): r > 0.9 very strong correlation; 0.7 < r < 0.89 strong correlation; 0.4 < r < 0.69 moderate correlation; 0.1 < r < 0.39 weak correlation, and r < 0.09 negligible correlation [55,56]. Pearson’s correlation test was performed with the software Prism v. 6.01 (GraphPad Inc., Boston, MA, USA).

## 5. Conclusions

Bacterial prostatitis is caused by some Gram-positive and Gram-negative bacterial strains that have developed resistance to conventional antibacterial drugs. In this work, the antibacterial spectrum of different extracts from two *Epilobium* species, i.e., *E. angustifolium* and *E. parviflorum*, and their major bioactive compound, oenothein B, was investigated with outstanding results, thus confirming the traditional uses of these herbs in the treatment of infectious disorders of the genito-urinary tract and supporting their application in pharmaceutical and nutraceutical products. The results demonstrated significant antimicrobial activity for both the Gram-positive and Gram-negative species tested, with MIC values ranging from 4 to 512 µg/mL, often comparable to levofloxacin, the conventional antibacterial drug used for the assays as a reference. This work also demonstrated the crucial importance of the purification process to enhance the content of bioactive compounds, especially that of oenothein B, and thus to improve the antibacterial properties of the extracts. These results suggest the outstanding potential of both *Epilobium* species as therapeutic alternatives or complementary agents to conventional antibiotic treatments of prostatitis and other urinary tract infections. However, it is crucial to acknowledge that the current study has been conducted in vitro, and further in vivo studies are necessary to confirm the observed antimicrobial activity in relevant animal models of prostatitis. Further studies are also needed to assess the possible synergistic action with conventional antibacterial drugs and to develop formulations to boost the already promising antibacterial potential of the extracts.

## Figures and Tables

**Table 1:** High-performance liquid chromatography–tandem mass spectrometry (HPLC-MS/MS) acquisition parameters, working in dynamic “multiple reaction monitoring” mode, including retention time (Rt) and delta retention time (ΔRt) for each optimized transition.

No.	Compound	Molecular Weight (g/mol)	Precursor Ion (*m*/*z*)	Product Ion (*m*/*z*)	Fragmentor (V)	Collision Energy (V)	Retention Time (Rt) (min)	Delta Retention Time (ΔRt)	Polarity
1	gallic acid	170	169	125	97	12	3.4	3	negative
2	chlorogenic acid	354	353	191 ^1^, 179	82	12, 12	5.6	3	negative
3	oenothein B	1569	783	783 ^1^, 765	40	0, 15	6.5	4	negative
4	myricetin-3-*O*-galactoside	480	479	316 ^1^, 271	170	24, 44	8.6	3	negative
5	myricitrin	464	463	316 ^1^, 271	155	24, 44	10.5	3	negative
6	quercetin-3-*O*-glucuronide	478	477	301 ^1^, 175	136	16, 16	10.7	3	negative
7	hyperoside	464	463	300 ^1^, 271	170	24, 48	10.7	3	negative
8	ellagic acid	302	301	301	170	0	11.8	4	negative
9	kaempferol-3-*O*-glucoside	448	447	284 ^1^, 255	170	24, 40	12.5	3	negative
10	kaempferol-3-*O*-glucuronide	462	461	285	126	16	12.7	3	negative
11	quercitrin	448	447	300 ^1^, 271	160	24, 44	12.9	3	negative
12	myricetin	318	317	179 ^1^, 193	150	16, 16	15.7	4	negative
13	kaempferol-3-*O*-rhamnoside	432	431	285 ^1^, 255	150	16, 30	15.3	3	negative
14	quercetin	302	301	151 ^1^, 179	145	16, 12	20.4	4	negative
15	kaempferol	286	285	285	160	0	25.1	4	negative

^1^ These product ions were used for quantification; the others to confirm the analytes.

**Table 2:** HPLC-MS/MS method validation parameters: regression equation, coefficient of determination (R^2^), limits of detection (LODs), limits of quantification (LOQs), and reproducibility for the fifteen monitored compounds.

					Repeatability (% RSD)	
Compound	Regression Equation	R^2^	LOD (µg/L)	LOQ (µg/L)	Intraday	Inter-Day
gallic acid	*y* = 11,732*x* + 1871.5	0.999	3	10	0.97	6.28
chlorogenic acid	*y* = 19,982*x* − 175.02	1	2	5	1.30	1.26
oenothein B	*y* = 2006.7*x* − 680.97	0.999	33	100	0.17	0.81
myricetin-3-*O*-galactoside	*y* = 19,522*x* − 960.3	0.999	2	5	0.76	12.73
myricitrin	*y* = 21,108*x* − 787.66	0.999	2	5	0.02	10.05
quercetin-3-*O*-glucuronide	*y* = 22,667*x* − 2599.7	0.999	3	10	0.35	11.85
hyperoside	*y* = 14,458*x +* 75.328	1	2	5	0.79	10.64
ellagic acid	*y* = 28,444*x* − 4135.5	0.999	17	50	4.67	3.09
kaempferol-3-*O*-glucoside	*y* = 16,461*x* − 538.22	1	2	5	0.42	12.25
kaempferol-3-*O*-glucuronide	*y* = 15,941*x* − 1100.1	0.999	3	10	0.45	7.89
quercitrin	*y* = 18,629*x* − 202.71	1	2	5	0.06	2.70
myricetin	*y* = 6212.1*x* − 699.29	0.999	3	10	2.92	1.28
kaempferol-3-*O*-rhamnoside	*y* = 18,286*x* − 431.18	1	2	5	2.94	7.56
quercetin	*y* = 10,745*x* − 39.87	1	3	10	5.29	4.82
kaempferol	*y* = 94,006*x* – 33,517	0.998	3	10	8.26	7.04

**Table 3:** Quantification of the 15 bioactive compounds (expressed as mg/g of dry weight extract) in the two *Epilobium* species extracts using HPLC-MS/MS analysis.

No.	Compounds	RE1*_ang_* ^1^	RE2*_ang_*	PE*_ang_* ^2^	RE1*_par_*	RE2*_par_*	PE*_par_*
1	gallic acid	13.03	1.76	0.71	12.08	4.23	1.58
2	chlorogenic acid	2.23	2.13	6.34	0.82	1.29	2.57
3	oenothein B	119.98	127.78	255.86	153.02	168.25	327.57
4	myricetin-3-*O*-galactoside	0.36	0.57	1.88	3.52	5.58	10.16
5	myricitrin	0.07	0.11	0.31	2.97	5.01	9.56
6	quercetin-3-*O*-glucuronide	15.22	12.57	42.08	0.03	0.05	0.10
7	hyperoside	2.01	2.98	6.09	0.57	0.93	1.90
8	ellagic acid	4.52	1.15	4.16	6.14	5.42	11.54
9	kaempferol-3-*O*-glucoside	0.27	0.32	0.83	0.04	0.07	0.12
10	kaempferol-3-*O*-glucuronide	1.63	1.85	5.42	n.d.	n.d.	n.d.
11	quercitrin	0.86	2.24	3.81	0.32	0.66	1.32
12	myricetin	n.d.	n.d.	0.06	0.21	0.55	0.79
13	kaempferol-3-*O*-rhamnoside	0.71	1.12	2.73	0.07	0.14	0.23
14	quercetin	0.09	0.09	0.44	0.07	0.22	0.15
15	kaempferol	n.d. ^3^	n.q. ^4^	0.02	0.01	0.04	n.q.
	total	161.05	154.74	330.80	179.94	192.50	367.66

^1,2^ RE, raw extract; PE, purified extract; *ang*, *Epilobium angustifolium*; *parv*, *Epilobium parviflorum*; ^3^ n.d., not detectable; ^4^ n.q. not quantifiable. Each sample was analyzed in triplicate (n = 3) and relative standard deviation (RSD) values were from 0.10% to 12.73%.

**Table 4:** Minimum inhibitory concentration (MIC, µg/mL) and minimum bactericidal concentration (MBC, µg/mL) of *Epilobium angustifolium* and *Epilobium parviflorum* extracts.

	RE1_ang_		RE2_ang_		PE_ang_		RE1_par_		RE2_par_		PE_par_		Oenothein B		Levofloxacin
Gram-Positive Strains	MIC	MBC	MIC	MBC	MIC	MBC	MIC	MBC	MIC	MBC	MIC	MBC	MIC	MBC	MIC
*E. faecalis* ATCC 29212	16	32	32	64	16	32	8	16	32	64	8	16	8	16	2
*E. faecalis* BS	16	32	64	128	16	32	16	32	32	64	8	16	8	16	8
*S. aureus* ATCC 25923	8	16	8	16	4	8	8	16	8	16	4	8	4	8	0.5
*S. aureus* ATCC 29213	8	16	8	16	4	8	8	16	8	16	4	8	4	8	0.5
*S. aureus* ATCC 43300	16	32	16	32	8	16	16	32	8	16	16	32	8	16	1
**Gram-negative strains**															
*E. coli* ATCC 25922	256	512	512	1024	256	512	256	512	512	1024	256	512	1024	2048	0.12
*E. coil* ATCC 35218	512	512	512	1024	512	1024	512	512	512	512	512	1024	1024	2048	0.12
*E. coli* BS	1024	2048	1024	2048	512	1024	1024	2048	1024	2048	512	1024	1024	2048	1
*K. pneumoniae* ATCC 13883	128	256	64	128	32	64	64	128	64	128	32	64	256	512	8
*K. pneumoniae* ATCC 70063	128	256	64	128	32	64	64	128	64	128	32	64	128	256	8
*K. pneumoniae* BS	128	256	128	256	32	64	64	128	128	256	32	64	64	128	32
*P. mirabilis* BS	128	256	64	128	32	64	128	256	128	256	32	64	128	256	8
*P. aeruginosa* ATCC 27853	64	128	16	32	8	16	32	64	32	64	8	16	64	128	4

RE1_ang_, *Epilobium angustifolium* raw dry extract (RE) (H_2_O); RE2_ang_, *Epilobium angustifolium* raw dry extract (EtOH); PE_ang_**,** *Epilobium angustifolium* purified dry extract (EtOH); RE1_par_ *Epilobium parviflorum* raw dry extract (H_2_O); RE2_par_, *Epilobium parviflorum* raw dry extract (EtOH); PE_par_, *Epilobium parviflorum* purified dry extract (EtOH).

**Table 5:** Pearson correlation analysis on the contribution of the target phenolic compounds to the antibacterial activity.

	*E. faecalis* ATCC 29212	*E. faecalis* BS	*S. aureus* ATCC 25923	*S. aureus* ATCC 29213	*S. aureus* ATCC 43300	*E. coli* ATCC 25922	*E. coil* ATCC 35218	*E. coli* BS	*K. pneumoniae* ATCC 13883	*K. pneumoniae* ATCC 70063	*K. pneumoniae* BS	*Proteus mirabilis* BS	*P. aeruginosa* ATCC 27853
gallic acid	NS ^1^	NS	NS	NS	NS	NS	NS	NS	NS	NS	NS	−0.817 *^,2^	−0.868 *
chlorogenic acid	NS	NS	NS	NS	NS	NS	NS	NS	NS	NS	NS	NS	NS
oenothein B	NS	NS	−0.938 **	−0.938 **	NS	NS	NS	−0.938 **	NS	NS	−0.802 *	NS	NS
myricetin-3-*O*-galactoside	NS	NS	NS	NS	NS	NS	NS	NS	NS	NS	NS	NS	NS
myricitrin	NS	NS	NS	NS	NS	NS	NS	NS	NS	NS	NS	NS	NS
quercetin-3-*O*-glucuronide	NS	NS	NS	NS	NS	NS	NS	NS	NS	NS	NS	NS	NS
hyperoside	NS	NS	NS	NS	NS	NS	NS	NS	NS	NS	NS	NS	NS
ellagic acid	NS	NS	NS	NS	NS	NS	NS	NS	NS	NS	NS	NS	NS
kaempferol-3-*O*-glucoside	NS	NS	NS	NS	NS	NS	NS	NS	NS	NS	NS	NS	NS
kaempferol-3-*O*-glucuronide	NS	NS	NS	NS	NS	NS	NS	NS	NS	NS	NS	NS	NS
quercitrin	NS	NS	NS	NS	NS	NS	NS	NS	NS	NS	NS	NS	NS
myricetin	NS	NS	NS	NS	NS	NS	NS	NS	NS	NS	NS	NS	NS
kaempferol-3-*O*-rhamnoside	NS	NS	NS	NS	NS	NS	NS	NS	NS	NS	NS	NS	NS
quercetin	NS	NS	NS	NS	−0.847 *	NS	NS	NS	NS	NS	NS	NS	NS
kaempferol	NS	NS	NS	NS	NS	NS	NS	NS	NS	NS	NS	NS	NS
total	NS	NS	−0.982 ***	−0.982 ***	NS	NS	NS	−0.982 ***	NS	NS	−0.802 *	NS	NS

^1^ NS, refer to not significant correlation; ^2^ the asterisk describes the test significance as follows: * for 0.05 < *p* < 0.01; ** 0.01 < *p* < 0.001 and *** for *p* < 0.001.

**Table 6:** Yields (% *w*/*w*) obtained for the extraction and purification processes.

	Yield (% *w*/*w*)
RE1*_ang_*	26.4
RE1*_par_*	26.9
RE2*_ang_*	26.9
RE2*_par_*	22.4
PE*_ang_*	10.0
PE*_par_*	9.0

RE, raw extract; PE, purified extract; ang, *Epilobium angustifolium*; parv, *Epilobium parviflorum*.

## Data Availability

All data are included in the manuscript.

## Supplementary Materials

The following supporting information can be downloaded at: https://www.mdpi.com/article/10.3390/antibiotics14020117/s1, Table S1: elution gradients employed for HPLC-MS/MS method development. Figure S1. Total ion chromatogram and dynamic “multiple reaction monitoring” mode chromatograms of single compounds of the RE2*_ang_*. Figure S2. Total ion chromatogram and dynamic “multiple reaction monitoring” mode chromatograms of single compounds of the RE2*_parv_*. Figure S3. Total ion chromatogram and dynamic “multiple reaction monitoring” mode chromatograms of single compounds of the PE*_ang_*. Figure S4. Total ion chromatogram and dynamic “multiple reaction monitoring” mode chromatograms of single compounds of the PE*_parv_*.
