# Supplementary material for: Efficacy of Willow Herb (Epilobium angustifolium L. and E. parviflorum Schreb.) Crude and Purified Extracts and Oenothein B Against Prostatic Pathogens
Source: Antibiotics (Basel). 2025 Jan 23;14(2):117. doi: 10.3390/antibiotics14020117 (PMC11851509; doi:10.3390/antibiotics14020117)
Supplement: Supplementary file 1 [file antibiotics-14-00117-s001.zip › antibiotics-3328092-supplementary.pdf]

Table S1. Different tested elution gradients using water (A) and acetonitrile (B) both with formic acid.

Gradient 1	Solvent (%)	
**Time (min)**	**A**	**B**
**0**	80	20
**2**	80	20
**22**	10	90
**25**	10	90
**33**	80	20
**Gradient 2**	**Solvent (%)**	
**Time (min)**	**A**	**B**
**0**	80	20
**2**	80	20
**22**	35	65
**25**	35	65
**31**	80	20
**Gradient 3**	**Solvent (%)**	
**Time (min)**	**A**	**B**
**0**	80	20
**2**	80	20
**30**	50	50
**32**	50	50
**37**	80	20
**Gradient 4**	**Solvent (%)**	
**Time (min)**	**A**	**B**
**0**	85	15
**30**	55	45
**32**	55	45
**37**	85	15

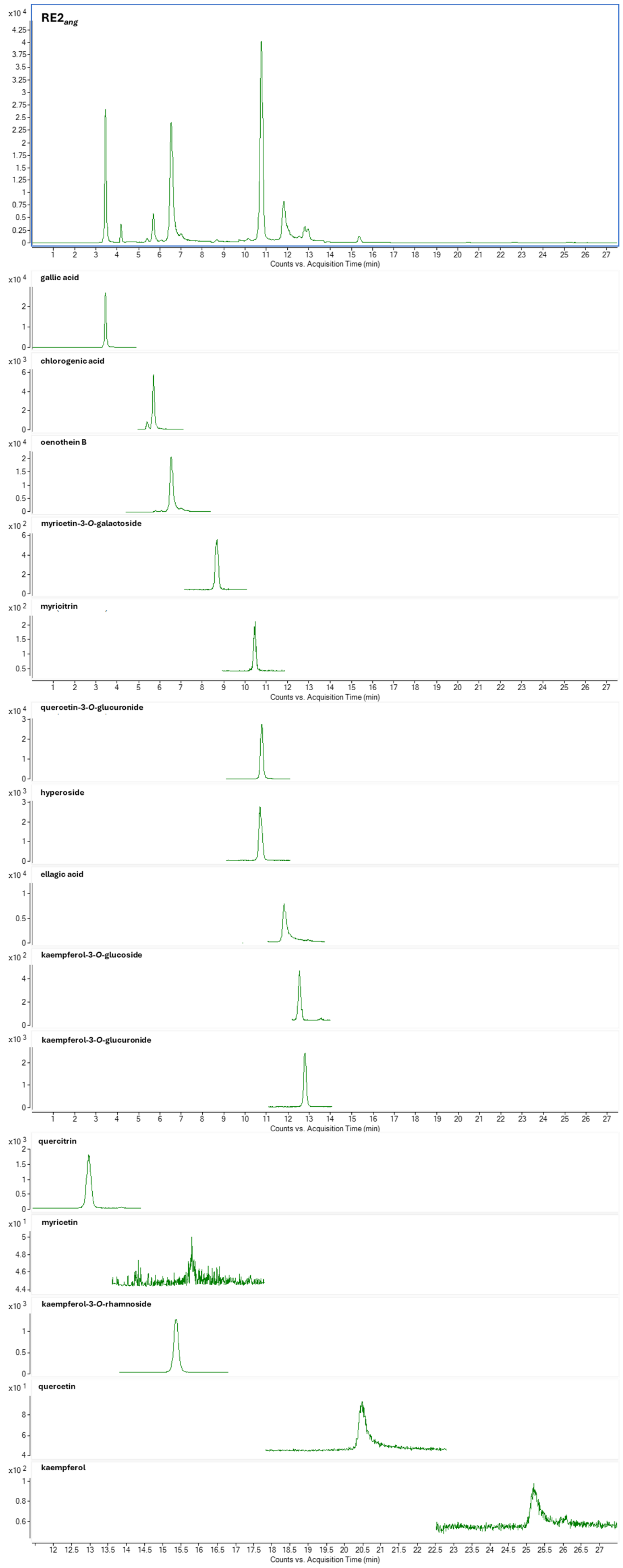

Figure S1. Total ion chromatogram and dynamic "multiple reaction monitoring" mode chromatograms of single compounds of the RE2*ang*.

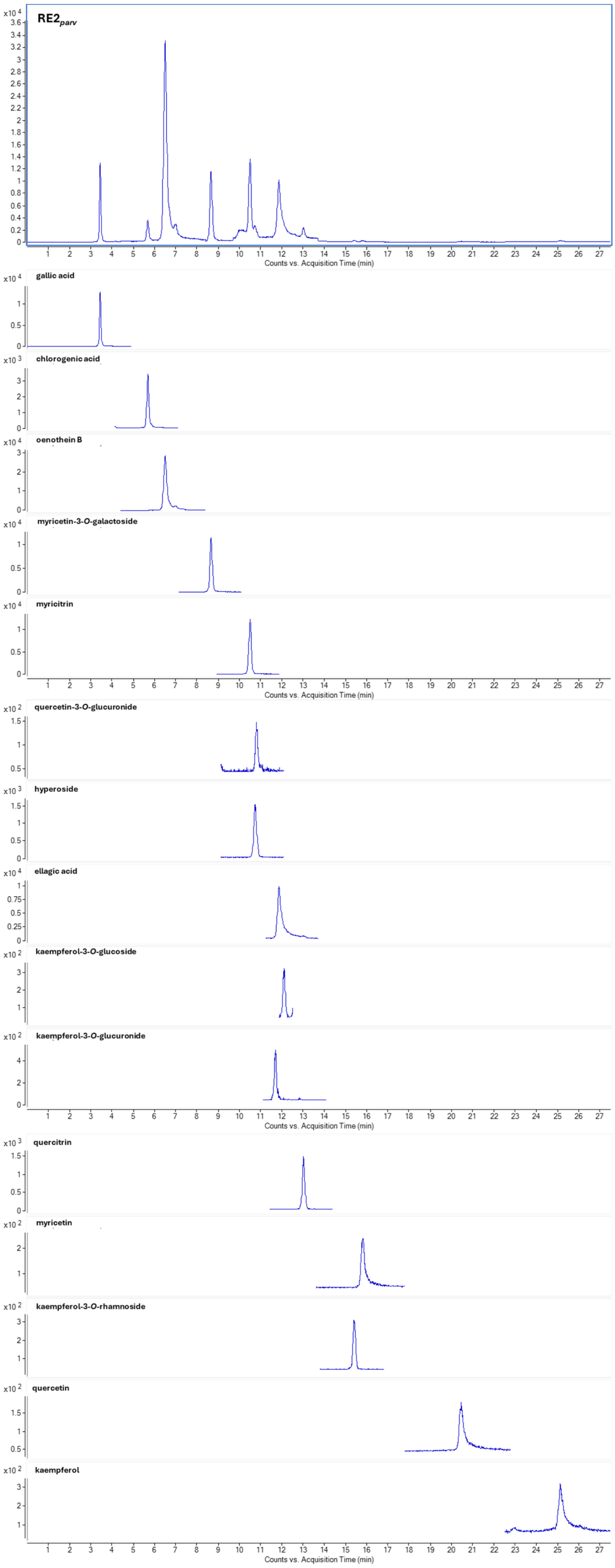

Figure S2. Total ion chromatogram and dynamic "multiple reaction monitoring" mode chromatograms of single compounds of the RE2$_{parv}$.

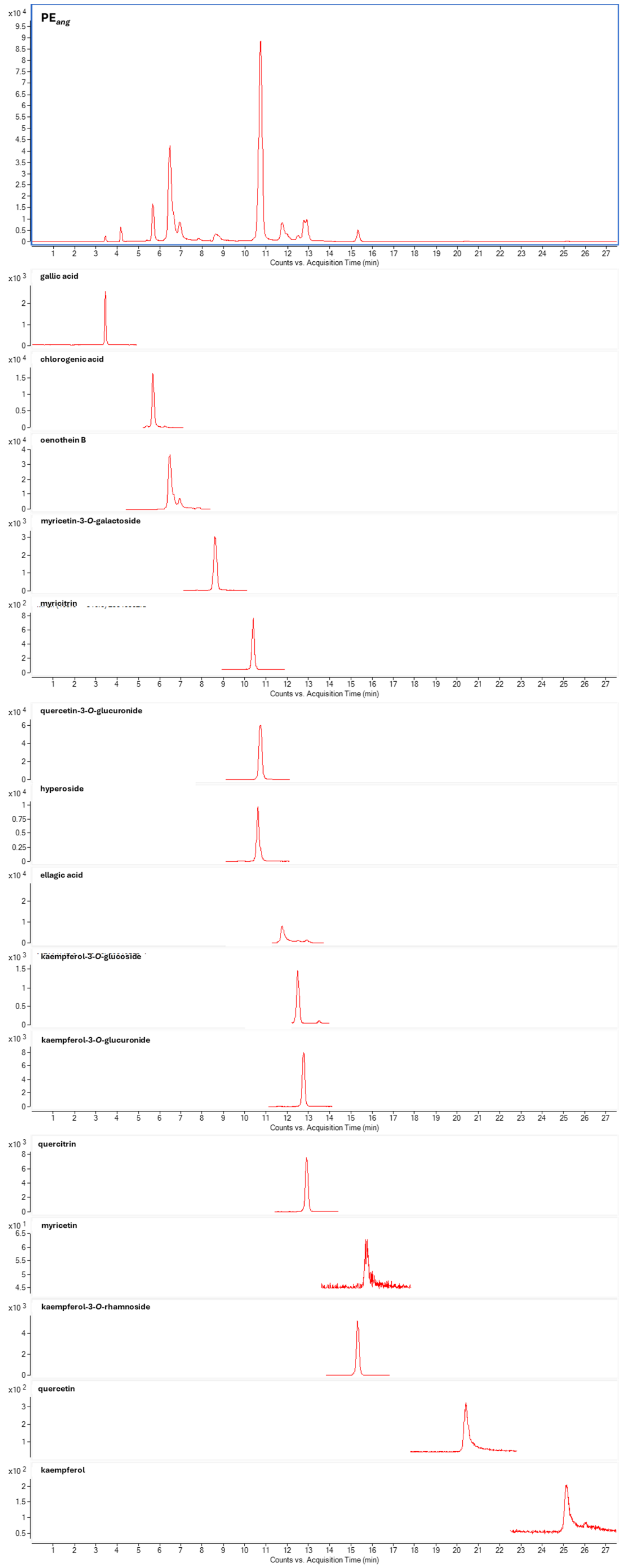

Figure S3. Total ion chromatogram and dynamic "multiple reaction monitoring" mode chromatograms of single compounds of the PE*ang*.

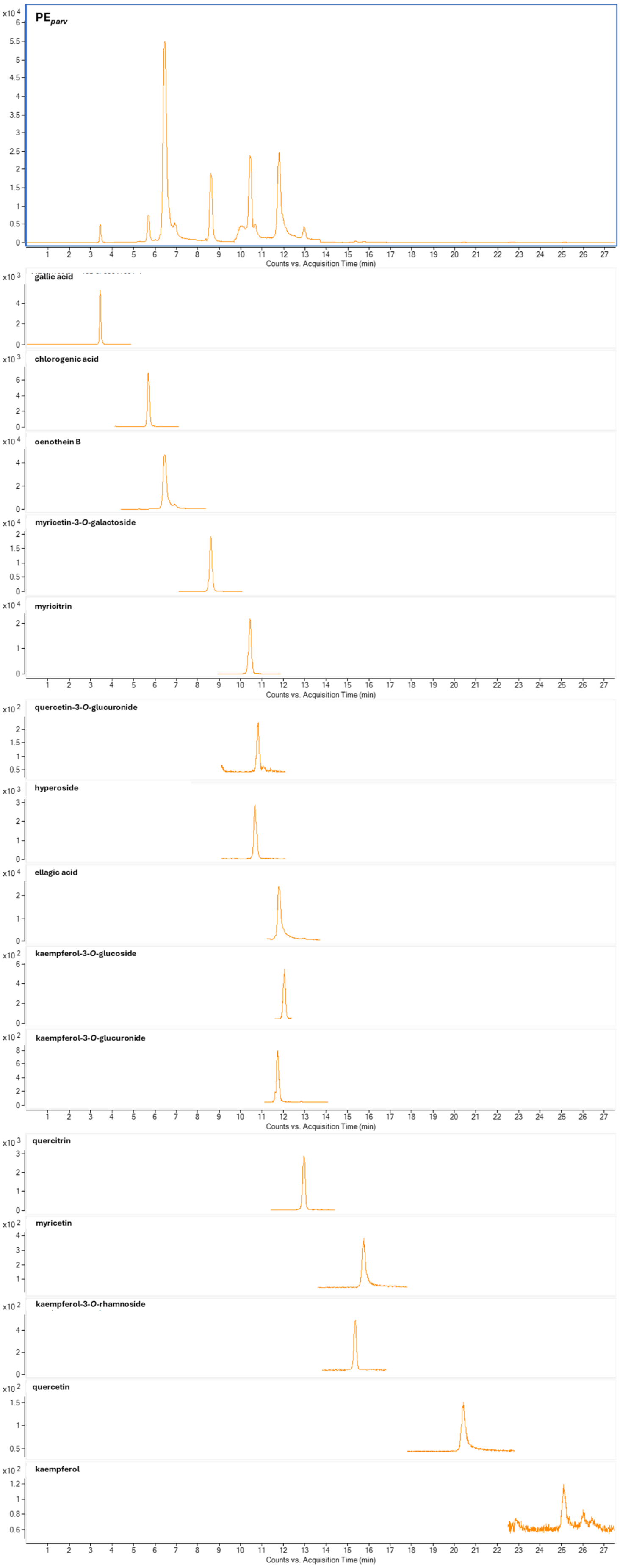

Figure S4. Total ion chromatogram and dynamic “multiple reaction monitoring” mode chromatograms of single compounds of the PE*parv*.
